# Gauge and unitary transformations in multipolar quantum optics

**DOI:** 10.1098/rsta.2023.0330

**Published:** 2024-12-24

**Authors:** Mohamed Babiker

**Affiliations:** ^1^ School of Physics, Engineering and Technology, University of York, England, YO10 5DD, UK

**Keywords:** QED, PZW Theory, multipolar quantum optics, Quantum Optics

## Abstract

Multipolar quantum optics deals with the interaction of light with matter as a many-body bound system of charged particles where the coupling to electromagnetic fields is in terms of the multipolar electric polarization and magnetization. We describe two transformations applied to the conventional non-relativistic formalism, namely a gauge transformation applied directly to the fields at the Lagrangian stage and a unitary transformation applied to the old Hamiltonian. We show how such transformations lead to the same Power–Zienau–Woolley (PZW) formulation of the quantum electrodynamics (QED) of an overall electrically neutral many-body bound system of charges, including the internal motion as well as the gross dynamics of the centre of mass. Besides highlighting the utility of the multipolar formalism as a reliable and convenient platform in dealing with optical processes in atomic and molecular physics, it is shown how the analysis can also lead to the identification of the Röntgen effect arising from the gross motion of an electric dipole moment in a magnetic field and the Aharonov–Casher effect due to the motion of a magnetic dipole moment in an electric field. The importance of the two effects is pointed out in both experimental and theoretical contexts.

This article is part of the theme issue ‘The quantum theory of light’.

## 1. Introduction

Applications of quantum electrodynamics (QED) involving the interactions of light with atomic and molecular matter often used the conventional minimal coupling formulation in which potentials have a central role in their description of the electromagnetic fields, and they require the specification of a gauge condition such as the Coulomb or the Lorenz gauge. In such treatments, applications of the perturbation theory often involve interaction terms which lack direct interpretation, i.e. whether it is an electric or a magnetic interaction one is dealing with. It then became clear that it may be advantageous if a formulation could be found in which the gauge-invariant electric and magnetic field variables can take the central role rather than the potentials. Furthermore, Power & Zienau [1] pointed out that the evaluation of radiative transitions using the Coulomb gauge led to ambiguities which would not arise if the electric and magnetic polarizations are the sources (rather than the charge and current densities), which couple to the electric and magnetic fields (rather than the potentials). Such a formulation would have the advantage of being manifestly gauge-invariant. This was the motivation by Power and Zienau in devising their 1959 theory. However, their theory then only dealt with electric dipole interactions and so involved the use of a canonical transformation applied to the conventional Hamiltonian in the dipole approximation. Power and Zienau’s work thus put the theory on a firm basis in terms of a canonical transformation and explored its consequences for actual processes [2], highlighting the calculational advantages of the transformed theory and agreement with experiment. Generalizations to include all multipoles in closed forms and for a many-body atomic and molecular system were later done by a number of authors [3–9]. Work by Woolley [10–13] led to an equivalent formulation of the multipolar theory of interaction of atoms and molecules with electromagnetic fields, and so, the theory is now known as the Power–Zienau–Woolley (PZW) theory.

In 1983, it was suggested that the PZW theory can be equivalently achieved using a gauge transformation [14], but although multipolar in treatment, it only dealt with the two-particle case and led to a new Lagrangian with the gauge transformation applied to the electromagnetic fields, and the new Lagrangian then led to the new ‘transformed’ Hamiltonian. The analytical framework based on this gauge transformation represents a valid platform for the description of the interacting system. However, the details are rather different from an equivalent canonical transformation which would start at the old Hamiltonian operator (derived from the old Lagrangian) on which a canonical transformation is performed to obtain new generalized momenta and a new Hamiltonian.

Our aim here is to describe the two transformations mentioned above and discuss their manifestations specifically in the context of the quantum optics of atoms and molecules [15,16]. In addition to emphasizing the advantages of using the multipolar formalism to evaluate processes in atomic and molecular physics, we also show how the analysis has led to the Röntgen effect [8,17–21] and the Aharonov–Casher effect [22–25], which are currently of interest.

The plan of this article is as follows. In §2, we define the many-body bound system of particles, typically forming an atom or a molecule, and present the conventional theory leading from the old Lagrangian to the old Hamiltonian via the canonical procedure. In §3 and §4, we consider the two transformations in turn focusing specifically on neutral atomic and molecular systems interacting with electromagnetic fields via their electric and magnetic multipoles. The two transformations, in effect, involve the same generating function defined as the space integral of the product of the electric polarization vector P with the vector potential 𝐀 [1]. §3 describes the formalism needed to generate the new Lagrangian arising from the gauge transformation and moves on to determine the new gauge Hamiltonian. The details are markedly different from the case of the canonical transformation in §4 in which we apply the unitary transformation involving the same generating function to derive the new canonical momenta, and we proceed to find the new Hamiltonian, which, we demonstrate, coincides with that arising from the gauge transformation. Either of the two identical Hamiltonians arising from the two transformations defines the Power–Zienau–Woolley theory which emerges as suitable for the many-body bound system. In §5, we apply the PZW theory to the electrically neutral two-particle case as for the hydrogenic atom. This constitutes a transparent illustration of the general case in which the Hamiltonian splits into well-defined terms whose interpretation is easy to comprehend, including the Röntgen interaction and the Aharonov–Casher interaction terms. Section 6 contains a summary and our conclusions, together with further comments.

## 2. Conventional QED

The many-body system consists of N particles of charges $e_{\alpha }:e_{1},e_{2},.....e_{N}$ of masses $m_{\alpha }:m_{1},m_{2},....m_{N}$ interacting with electromagnetic fields. We develop the theory from first principles for this case, beginning with the conventional non-relativistic minimal coupling Lagrangian for the above system of charges in interaction with electromagnetic potentials 𝐀 and Φ. We write


(2.1) $$\begin{array}{lcr} & L=\frac{1}{2}\sum_{\alpha =1}^{N}m_{\alpha }{\dot{𝐪}}_{\alpha }^{2}+\int L(𝐫)\;d^{3}𝐫, & \end{array}$$

where L is the Lagrangian density


(2.2) $$\begin{array}{lcr} & L(𝐫)=\frac{1}{2}\epsilon_{0}\left\{(\dot{𝐀}+\nabla \Phi {)}^{2}-c^{2}(\nabla \times 𝐀{)}^{2}\right\}+𝐉(𝐫)\cdot 𝐀(𝐫)-\rho (𝐫)\Phi (𝐫). & \end{array}$$

Here ${𝐪}_{\alpha },\;\alpha =1,2,..N$ are the particle position vectors. The charge and current densities receive contributions from all charged particles and are given by


(2.3) $$\begin{array}{lcr} & \rho (𝐫)=\sum_{\alpha =1}^{N}e_{\alpha }\delta (𝐫-{𝐪}_{\alpha });\;\;\;𝐉(𝐫)=\sum_{\alpha =1}^{N}e_{\alpha }{\dot{𝐪}}_{\alpha }\delta (𝐫-{𝐪}_{\alpha }). & \end{array}$$

The equations of motion arising from the Euler–Lagrange equations are the source Maxwell’s equations


(2.4) $$\begin{array}{lcr} & \nabla \cdot 𝐄=\rho /\epsilon_{0};\;\;\;\nabla \times 𝐁=\mu_{0}\epsilon_{0}\dot{𝐄}+\mu_{0}𝐉, & \end{array}$$

and Newton’s law with the Lorentz force for each of the constituent particles


(2.5) $$m_{\alpha }{\ddot{q}}_{\alpha }=e_{\alpha }E(q_{\alpha })+e_{\alpha }{\dot{q}}_{\alpha }\times B(q_{\alpha }),$$

where 𝐄 and 𝐁 are defined in the usual fashion


(2.6) $$\begin{array}{lcr} & 𝐄=-\dot{𝐀}-\nabla \Phi ;\;\;\;𝐁=\nabla \times 𝐀. & \end{array}$$

As we shall see below, it turns out, conveniently, that theory can avoid the explicit appearance of the scalar potential Φ as a dynamical variable in favour of the longitudinal component of the vector potential. We then adopt ${𝐪}_{\alpha },\;\alpha =1,2,..N$ and 𝐀 as the dynamical variables. The corresponding canonical momenta are


(2.7) $$\begin{array}{lcr} & {𝐩}_{\alpha }=\frac{\partial L}{\partial {\dot{𝐪}}_{\alpha }}=m_{\alpha }{\dot{𝐪}}_{\alpha }+e_{\alpha }𝐀({𝐪}_{\alpha }) & \end{array}$$

and


(2.8) $$\Pi (r)=\frac{\partial L}{\partial \dot{A}}=\epsilon_{0}\left\{\dot{A}(r)+\nabla \Phi (r)\right\}=-\epsilon_{0}E(r).$$

The corresponding Hamiltonian is


(2.9) $$\begin{array}{lcr} & H=\sum_{\alpha }{𝐩}_{\alpha }{.\dot{𝐪}}_{\alpha }+\int d^{3}𝐫\;\Pi \cdot \dot{𝐀}\;-L, & \end{array}$$

which gives


(2.10) $$H=\sum_{\alpha }\frac{{\left\{p_{\alpha }-e_{\alpha }A(q_{\alpha })\right\}}^{2}}{2m_{\alpha }}+\frac{1}{2}\int d^{3}r\left(\frac{\Pi^{2}}{\epsilon_{0}}+\frac{B^{2}}{\mu_{0}}\right)+\int d^{3}r\left(\nabla .\Pi +\rho \right)\Phi .$$

We may now remove the final term in the above Hamiltonian by direct use of equation (2.8), together with equation (2.4). We get


(2.11) $$\begin{array}{lcr} & H=\sum_{\alpha }\frac{{\left\{{𝐩}_{\alpha }-e_{\alpha }𝐀({𝐪}_{\alpha })\right\}}^{2}}{2m_{\alpha }}+\frac{1}{2}\int d^{3}𝐫\left(\frac{\Pi^{2}}{\epsilon_{0}}+\frac{{𝐁}^{2}}{\mu_{0}}\right). & \end{array}$$

The commutation relations are


(2.12) $$\begin{array}{lcr} & [p_{\alpha_{i}},q_{\beta_{j}}]=-i\hbar \delta_{\alpha \beta }\delta_{ij};\;\;\;[\Pi_{i}(𝐫)\;,\;A_{j}({𝐫}^{\prime })]=-i\hbar \delta_{ij}\delta (𝐫-{𝐫}^{\prime }). & \end{array}$$

The final form of the Hamiltonian can be written entirely in terms of gauge invariant variables ${\dot{𝐪}}_{\alpha },𝐄$ and 𝐁


(2.13) $$\begin{array}{lcr} & H=\frac{1}{2}\sum_{\alpha }m_{\alpha }{\dot{𝐪}}_{\alpha }^{2}+\frac{1}{2}\epsilon_{0}\int d^{3}𝐫\left\{{𝐄}^{2}+c^{2}{𝐁}^{2}\right\}. & \end{array}$$

This is the universal form of the Hamiltonian to which all forms arising via transformations must be reduced.

### (a) Electric polarization and centre of mass

To be able to proceed to a multipolar formulation, we need to identify a reference centre, and we choose the centre of mass coordinate for this purpose relative to which the particles’ coordinates are referred. The idea is an attempt to separate the internal motion from the gross motion of the centre of mass. This separation cannot unfortunately be done straightforwardly on the conventional theory represented by the Hamiltonian equation (2.11) since the fields enter the formalism directly in terms of the particle coordinates ${𝐪}_{\alpha }$. It turns out that the separation is best achieved in the multipolar formulation as we now explain.

The centre of mass coordinate is $𝐑=\frac{\sum_{\alpha }m_{\alpha }{𝐪}_{\alpha }}{M}$, where M is the total mass of the system of charges $M=\sum_{\alpha }m_{\alpha }.$ As we pointed out earlier, our concern here is with a version of theory which describes both the internal and translational motions of the system of charges in the presence of electromagnetic fields. The translational motion is appropriately described in terms of the centre of mass which, conveniently, also provides a natural choice for the point in space (albeit not a fixed point) relative to which multipolar moments are defined. Relative to the centre of mass, the electric polarization vector field of the charge system is written in the form


(2.14) $$P(r,\{q_{\alpha }\})=\sum_{\alpha }e_{\alpha }\int_{0}^{1}d\lambda (q_{\alpha }-R)\delta \;\left\{r-R-\lambda (q_{\alpha }-R)\right\}.$$

Note that P contains contributions from all the particles, of position variables $\left\{{𝐪}_{\alpha }\right\}$ including the nucleus, and that the centre of mass is not fixed in space. Because of the motion of the constituents, the centre of mass possesses a velocity $\dot{𝐑}$. The division of the motion into internal plus translational motions must be carried out in such a way that no additional degrees of freedom are introduced in the theory. This form of the electric polarization field vector conforms with the requirement that [14]


(2.15) $$\begin{array}{lcr} & \nabla \cdot P=-\rho , & \end{array}$$

where ρ is the electric charge density of the N point charges, as given in equation (2.3). The physical meaning of equation (2.15) can be seen when both sides of the equation are integrated over a space of volume V enclosed by a surface S. The right-hand side then yields the total charge −Q inside V, while the left-hand side can be converted into a surface integral over S using Gauss’ theorem, which is the integration over the surface S of outward normal component of the Polarization field. This is then equal to the negative of the total charge within V. In our case of a set of discrete point charges, the proof of equation (2.15) can be done straightforwardly as a generalization of the two-particle case (see Appendix B of [14]) to N-particles.

## 3. Gauge transformations

The gauge transformation we are concerned with here is specific to the electromagnetic interactions with bound charged particles such as atoms and molecules. In electrodynamics, the presence of charges involves a minimal coupling Lagrangian L in terms of a vector potential 𝐀 and a scalar Φ. The application of a gauge transformation simply changes the electromagnetic potentials to new ones involving a generating functional $\tilde{S}$ [14,26] as follows.


(3.1) $$A\to A^{\prime }=A-\nabla \tilde{S};\;\;\;\Phi \to \Phi^{\prime }=\Phi +\frac{\partial \tilde{S}}{\partial t}.$$

As is always the case with gauge transformations in electromagnetism, the generating function is a scalar function. Here it is defined as follows:


(3.2) $$\begin{array}{lcr} & \tilde{S}({𝐫}^{\prime })=\int d^{3}𝐫\;𝐀(𝐫)\cdot F(𝐫,{𝐫}^{\prime }), & \end{array}$$

with F given by


(3.3) $$\begin{array}{lcr} & F(𝐫,{𝐫}^{\prime })=\int_{0}^{1}d\lambda ({𝐫}^{\prime }-𝐑)\delta \left\{𝐫-𝐑-\lambda ({𝐫}^{\prime }-𝐑)\right\}. & \end{array}$$

Note that the the gauge function is related to the generating function S as follows:


(3.4) $$\begin{array}{lcr} & \sum_{\alpha }e_{\alpha }\tilde{S}({𝐫}^{\prime }={𝐪}_{\alpha })=\sum_{\alpha }e_{\alpha }\int d^{3}𝐫\;𝐀(𝐫)\cdot F(𝐫,{𝐫}^{\prime }={𝐪}_{\alpha })=\sum_{\alpha }S_{\alpha }=S=\int d^{3}𝐫\;𝐀(𝐫)\cdot P(𝐫,\{{𝐪}_{\alpha }\}), & \end{array}$$

where $P(𝐫,\{{𝐪}_{\alpha }\})=\sum_{\alpha }e_{\alpha }F(𝐫,{𝐫}^{\prime }={𝐪}_{\alpha })$ is the polarization field as in equation (2.14). Applying the gauge transformation equation (3.1) to the old Lagrangian equations (2.1) and (2.2), we obtain the transformed Lagrangian as follows:


(3.5) $$L^{\prime }=L-\int d^{3}r\left\{J\cdot \nabla \tilde{S}+\rho \frac{\partial \tilde{S}}{\partial t}\right\}.$$

Recall that 𝐉 and ρ are given by


(3.6) $$J=\sum_{\alpha }e_{\alpha }{\dot{q}}_{\alpha }\delta (r-q_{\alpha });\;\;\;\;\rho =\sum_{\alpha }e_{\alpha }\delta (r-q_{\alpha }).$$

Substituting for 𝐉 and ρ, we have


(3.7) $$\int d^{3}rJ\cdot \nabla \tilde{S}=\int d^{3}r\sum_{\alpha }e_{\alpha }{\dot{q}}_{\alpha }\delta (r-q_{\alpha })\cdot \nabla \int d^{3}r^{\prime }\;A(r^{\prime })\cdot F(r,r^{\prime }).$$

Integrating over 𝐫 and so replacing 𝐫 with ${𝐪}_{\alpha }$, the expression can then be rearranged as follows:


(3.8) $$\begin{array}{rl} \int d^{3}rJ\cdot \nabla \tilde{S} & =\sum_{\alpha }e_{\alpha }{\dot{q}}_{\alpha }\cdot \nabla^{\alpha }\int d^{3}r^{\prime }\;A(r^{\prime })\cdot F(q_{\alpha },r^{\prime })\\ & =\sum_{\alpha }e_{\alpha }{\dot{q}}_{\alpha }\cdot \nabla^{\alpha }\int d^{3}r^{\prime }\;A(r^{\prime })\cdot F(q_{\alpha },r^{\prime })\\ & =\sum_{\alpha }{\dot{q}}_{\alpha }\cdot \nabla^{\alpha }S_{\alpha }. \end{array}$$

Evaluation of $\nabla^{\alpha }S_{\alpha }=e_{\alpha }\nabla^{\alpha }\int d^{3}{𝐫}^{\prime }\;𝐀({𝐫}^{\prime })\cdot F({𝐪}_{\alpha },{𝐫}^{\prime })$ yields


(3.9) $$\begin{array}{lcr} & \nabla^{\alpha }S_{\alpha }=e_{\alpha }𝐀({𝐪}_{\alpha })+\int d^{3}𝐫\;\Theta_{\alpha }(𝐫)\times 𝐁(𝐫), & \end{array}$$

where $\Theta_{\alpha }$ is given by


(3.10) $$\begin{array}{lcr} & \Theta_{\alpha }=\sum_{\beta }e_{\beta }\int_{0}^{1}d\lambda \left(\lambda \delta_{\alpha \beta }-\frac{m_{\alpha }}{M}(\lambda -1)\right)({𝐪}_{\beta }-𝐑)\;\delta \left\{𝐫-𝐑-\lambda ({𝐪}_{\beta }-𝐑)\right\}. & \end{array}$$

Consider next the last term in equation (3.5). We have


(3.11) $$-\int d^{3}r\rho \frac{\partial \tilde{S}}{\partial t}=-\int d^{3}r\sum_{\alpha }e_{\alpha }\delta (r-q_{\alpha })\dot{A}\cdot F(r,r^{\prime }).$$

Once again using $\dot{A}=-E-\nabla \Phi$ and that ∇⋅P=−ρ, we have


(3.12) $$\begin{array}{lcr} & -\int d^{3}𝐫\rho \frac{\partial \tilde{S}}{\partial t}=\int d^{3}𝐫[P\cdot 𝐄-\rho \Phi ]. & \end{array}$$

Substituting and after cancellations of the 𝐀 and Φ terms, the transformed Lagrangian emerges as follows:


(3.13) $$L^{\prime }=\sum_{\alpha =1}^{N}\left\{\frac{1}{2}m_{\alpha }{\dot{q}}_{\alpha }^{2}+{\dot{q}}_{\alpha }\cdot \int d^{3}r\;\Theta_{\alpha }(r)\times B(r)\right\}+\int d^{3}r\left[\frac{1}{2}\left\{\epsilon_{0}(E^{2}-c^{2}B^{2})\right\}+P\cdot E\right].$$

We may now evaluate the canonical momenta. We have, for the particles,


(3.14) $${p^{\prime }}_{\alpha }=\frac{\partial L^{\prime }}{\partial {\dot{q}}_{\alpha }}=m_{\alpha }{\dot{q}}_{\alpha }+\int d^{3}r\;\Theta_{\alpha }(r)\times B(r),$$

and for the fields we are concerned with momentum density


(3.15) $$\Pi^{\prime }=\frac{\partial L^{\prime }}{\partial \dot{A}}=\epsilon_{0}\left(\dot{A}+\nabla \Phi \right)-P\;=\;-\epsilon_{0}E-P,$$

and we note that that $\Pi^{\prime }$ is transverse since $\nabla \cdot \Pi^{\prime }=0$. The new Hamiltonian is


(3.16) $$H_{gauge}^{\prime }=\sum_{\alpha }{p^{\prime }}_{\alpha }\cdot {\dot{q}}_{\alpha }+\int d^{3}r\Pi^{\prime }\cdot \dot{A}-L^{\prime }.$$

It is straightforward to show that on substituting for the canonical momenta and the new Lagrangian, together with the use of $\dot{𝐀}=-𝐄-\nabla \Phi$ and $\nabla \cdot (\epsilon_{0}𝐄+P)=0$ and all-space integration by parts, we obtain the following form of the new Hamiltonian:


(3.17) $$H_{gauge}^{\prime }=\frac{1}{2}\sum_{\alpha }m_{\alpha }{\dot{q}}_{\alpha }^{2}+\frac{1}{2}\epsilon_{0}\int d^{3}r\left\{E^{2}+c^{2}B^{2}\right\}.$$

This looks exactly the same as the Hamiltonian of the conventional theory. However, and despite appearances, the new Hamiltonians will look different from the old as soon as we express the different terms in terms of the canonical momenta. That all Hamiltonians in this context must have the same universal forms was first pointed out in reference [14]. We expect the new Hamiltonian arising from the unitary transformation (discussed next) to reduce to this universal form. The useful form of the Hamiltonian is obtained when expressed in terms of the canonical momenta, which is


(3.18) $$\begin{array}{lcr} & H_{gauge}^{\prime }=\sum_{\alpha }\frac{{\left({{𝐩}^{\prime }}_{\alpha }+\int d^{3}𝐫\;\Theta_{\alpha }\times 𝐁(𝐫)\right)}^{2}}{2m_{\alpha }}+\frac{1}{2}\int d^{3}𝐫\;\left\{\frac{(\Pi^{\prime }+P{)}^{2}}{\epsilon_{0}}+\frac{{𝐁}^{2}}{\mu_{0}}\right\}. & \end{array}$$

The commutation relations of the particle and field variables are as follows:


(3.19) $$[p_{\alpha_{i}}^{\prime },q_{\beta_{j}}]=-i\hbar \delta_{\alpha \beta }\delta_{ij};\;\;\;\;\;\;[\Pi_{i}^{\prime }(r)\;,\;A_{j}(r^{\prime })]=-i\hbar \delta_{ij}^{⊥}(r-r^{\prime }),$$

where the transverse delta function $\delta_{ij}^{⊥}(𝐫-{𝐫}^{\prime })$ is as in [2]. Note that the commutation relation involving $\Pi^{\prime }$ and 𝐀 indicates that in a field quantization $\Pi^{\prime }$ is interpreted as the electric displacement field (for a discussion of the roles of the canonical field momenta in the free field quantization in the different formulations see [14]). The task is now complete with the determination of the transformed Hamiltonian and the commutation relations using the gauge transformation. As we shall see, this turns out to be identical to the outcome of the canonical transformation and is essentially the PZW theory.

## 4. Canonical transformation and the PZW theory

Canonical transformations are applicable as operator transformations at the Hamiltonian level. The field variables and the corresponding momenta are thus known in advance and arise from some Lagrangian functional in the usual manner. At the Hamiltonian level, it is possible to assign state vectors $|\Psi^{\prime }\rangle$ and a set of Hermitian operators, represented by O, corresponding to the observables of the system. We define a unitary operator U satisfying


(4.1) $$\begin{array}{lcr} & UU^{†}=U^{†}U=1. & \end{array}$$

We require U to act on the system by changing the state vectors to $|\Psi^{\prime }\rangle$ and the operators to $O^{\prime }$ including the system Hamiltonian which changes from H to $H^{\prime }$. The transformation equations are


(4.2) $$|\psi^{\prime }\rangle =U|\psi \rangle \;\;\;O^{\prime }=U\;O\;U^{†}.$$

It turns out that the unitary operator U has the form


(4.3) $$\begin{array}{lcr} & U=e^{iS}, & \end{array}$$

where *S* is the generating function. For the above canonical transformation to be equivalent to the gauge transformation, we must have the same form of S. The transformation makes use of the operator identity


(4.4) $$\begin{array}{lcr} & e^{A}Be^{-A}=B+[A,B]+\frac{1}{2}[A,[A,B]]+........, & \end{array}$$

with A=iS and B a system operator. All expectation values are unaffected


(4.5) $$\begin{array}{lcr} & \left\langle \psi^{\prime }|\;O^{\prime }\;|\psi^{\prime }\rangle =\langle \psi |U^{†}\;UOU^{†}\;U|\psi \rangle =\langle \psi |\;O\;|\psi \right\rangle & \end{array}$$

and all canonical commutation relations are preserved by such unitary transformations.

The new formulation is called the unitary equivalent of the original one. As pointed out earlier, in the context of quantum optics of atoms and molecules, the most prominent canonical transformation is the Power & Zienau transformation [2,27] which was further extended to the multipolar many-body form [3,6,7,28].

The procedure for casting the Hamiltonian into a form exhibiting independent translational and internal motions plus interactions depends on a first step on the application of a PZW transformation whose generating function is


(4.6) $$\begin{array}{lcr} & S(\{{𝐪}_{\alpha }\})=\int d^{3}𝐫𝐀(𝐫)\cdot P(𝐫;\{{𝐪}_{\alpha }\}). & \end{array}$$

Substituting for P, we have


(4.7) $$\begin{array}{lcr} & S(\{{𝐪}_{\alpha }\})=\sum_{\alpha }e_{\alpha }\int d^{3}𝐫\int_{0}^{1}d\lambda ({𝐪}_{\alpha }-𝐑)\cdot 𝐀(𝐫)\;\delta \left\{𝐫-𝐑-\lambda ({𝐪}_{\alpha }-𝐑)\right\}. & \end{array}$$

The direct route to the transformed theory is by using the operator identity, equation (4.4), together with the commutation rules in equation (2.12) to obtain new canonical momenta. The momentum conjugate to ${𝐪}_{\alpha }$ is denoted ${{𝐩}^{\prime }}_{\alpha }$. We have


(4.8) $$\begin{array}{lcr} & {{𝐩}^{\prime }}_{\alpha }=m_{\alpha }{\dot{𝐪}}_{\alpha }+e_{\alpha }𝐀({𝐪}_{\alpha })-\nabla^{\alpha }S, & \end{array}$$

where $\nabla^{\alpha }$ denotes differentiation with respect to the coordinates of ${𝐪}_{\alpha }$. The new momentum density conjugate to 𝐀 is denoted $\Pi^{\prime }$ which emerges in the form


(4.9) $$\begin{array}{lcr} & \Pi^{\prime }(𝐫)=\epsilon_{0}\left(\dot{𝐀}+\nabla \phi \right)-P\;=\;-\epsilon_{0}𝐄-P. & \end{array}$$

On taking the divergence of both sides and making use of the first Maxwell’s equation in equation (2.4), we find that $\Pi^{\prime }$ is transverse, i.e. $\nabla \cdot \Pi^{\prime }=0$. Evaluation of $\nabla^{\alpha }S$ yields


(4.10) $$\begin{array}{lcr} & \nabla^{\alpha }S=e_{\alpha }𝐀({𝐪}_{\alpha })+\int d^{3}𝐫\;\Theta_{\alpha }(𝐫)\times 𝐁(𝐫), & \end{array}$$

where $\Theta_{\alpha }$ is given by


(4.11) $$\begin{array}{lcr} & \Theta_{\alpha }=\sum_{\beta }e_{\beta }\int_{0}^{1}d\lambda \left(\lambda \delta_{\alpha \beta }-\frac{m_{\alpha }}{M}(\lambda -1)\right)({𝐪}_{\beta }-𝐑)\;\delta \left\{𝐫-𝐑-\lambda ({𝐪}_{\beta }-𝐑)\right\}. & \end{array}$$

Like the contributions of each particle to the electric polarization field, the vector $\Theta_{\alpha }$ depends only on the internal coordinates. An interesting sum rule that can be verified at once with the use of $\sum m_{\alpha }=M$ is the following:


(4.12) $$\begin{array}{lcr} & \sum_{\alpha }\Theta_{\alpha }=P. & \end{array}$$

Expressing the old Hamiltonian equation (2.11) in terms of the new canonical momenta ${{𝐩}^{\prime }}_{\alpha }$ and $\Pi^{\prime }$ and so eliminating the old momenta, we have


(4.13) $$\begin{array}{lcr} & H_{canonical}^{\prime }=\sum_{\alpha }\frac{{\left({{𝐩}^{\prime }}_{\alpha }+\int d^{3}𝐫\;\Theta_{\alpha }\times 𝐁(𝐫)\right)}^{2}}{2m_{\alpha }}+\frac{1}{2}\int d^{3}𝐫\;\left\{\frac{(\Pi^{\prime }+P{)}^{2}}{\epsilon_{0}}+\frac{{𝐁}^{2}}{\mu_{0}}\right\}. & \end{array}$$

This is identical to $H_{gauge}^{\prime }$, equation (3.18). The commutation relations of the particle and field variables are as before


(4.14) $$\begin{array}{lcr} & [p_{\alpha_{i}}^{\prime },q_{\beta_{j}}]=-i\hbar \delta_{\alpha \beta }\delta_{ij};\;\;\;\;\;\;[\Pi_{i}^{\prime }(𝐫)\;,\;A_{j}({𝐫}^{\prime })]=-i\hbar \delta_{ij}^{⊥}(𝐫-{𝐫}^{\prime }), & \end{array}$$

where the transverse delta function $\delta_{ij}^{⊥}(𝐫-{𝐫}^{\prime })$ is as defined in [2]. Note that here too the new Hamiltonian can be written in the universal form


(4.15) $$\begin{array}{lcr} & H_{canonical}^{\prime }=\frac{1}{2}\sum_{\alpha }m_{\alpha }{\dot{𝐪}}_{\alpha }^{2}+\frac{1}{2}\epsilon_{0}\int d^{3}𝐫\left\{{𝐄}^{2}+c^{2}{𝐁}^{2}\right\}, & \end{array}$$

as it should be. Of course, it is the canonical form in equation (4.13) which forms the starting point for evaluations of processes involving atoms and molecules in electromagnetic fields.

The multipolar Hamiltonian equation (4.13) differs from the conventional Hamiltonian in that the dependence on the particle variables ${𝐪}_{\alpha }$ now enters relative to the centre of mass coordinate with multipoles depending on ${𝐪}_{\alpha }-𝐑$. This feature greatly facilitates the division of the motion into internal and translational motions as we next discuss.

## 5. The two-particle case

If we consider two particles, only the formalism as given above is still valid except that all sums over the particle label α now range between 1 and 2. Since N=2, we have


(5.1) $$R=\frac{m_{1}q_{1}+m_{2}q_{2}}{M};\;\;\;M=m_{1}+m_{2},$$

and there will be only one internal variable 𝐪


(5.2) $$\begin{array}{lcr} & 𝐪={𝐪}_{1}-{𝐪}_{2}. & \end{array}$$

Using these relations, we obtain


(5.3) $$\begin{array}{lcr} & {𝐪}_{1}-𝐑=\frac{m_{2}}{M}𝐪;\;\;\;{𝐪}_{2}-𝐑=-\frac{m_{1}}{M}𝐪. & \end{array}$$

It can now be easily seen for the present two-particle case that the polarization equation ( (2.14) becomes a function of one internal variable 𝐪, and it exhibits no explicit dependence on the centre of mass variable 𝐑.

We are now in a position to eliminate the particle variables and momenta in favour of the above internal and centre of mass coordinates. We need to express ${𝐩}_{\alpha }^{\prime }$ in terms of the total momentum $𝐏=M\dot{𝐑}$ and the internal momentum 𝐩. We have for the momenta [29]


(5.4) $$\begin{array}{lcr} & {{𝐩}^{\prime }}_{\alpha }=\frac{m_{\alpha }}{M}𝐏+(-1{)}^{\alpha +1}𝐩;\;\;\;\alpha =1,2. & \end{array}$$

Substituting for ${{𝐩}^{\prime }}_{\alpha }$ in the Hamiltonian (remembering that we are really dealing with a two-particle system), we get


(5.5) $$\begin{array}{lcr} & H^{\prime }=\sum_{\alpha =1}^{2}\frac{{\left\{\frac{m_{\alpha }}{M}𝐏+(-1{)}^{\alpha +1}𝐩+\int d^{3}𝐫\Theta_{\alpha }\times 𝐁(𝐫)\right\}}^{2}}{2m_{\alpha }}+\frac{1}{2}\int d^{3}𝐫\left(\frac{(\Pi^{\prime }+P{)}^{2}}{\epsilon_{0}}+\frac{{𝐁}^{2}}{\mu_{0}}\right). & \end{array}$$

We can now expand the square in the first term and in the field term divide the polarization vector into separate transverse ⊥ and longitudinal ∥ components [2] to obtain a result which we can conveniently rearrange as follows:


(5.6) $$\begin{array}{rl} H^{\prime } & =\sum_{\alpha }\frac{P^{2}}{2M^{2}}m_{\alpha }+p^{2}\sum_{\alpha }\frac{1}{2m_{\alpha }}+\frac{1}{2\epsilon_{0}}\int (P^{∥}{)}^{2}d^{3}r+\frac{1}{2}\int d^{3}r\left\{\frac{{\Pi^{\prime }}^{2}}{\epsilon_{0}}+\frac{B^{2}}{\mu_{0}}\right\}\\ & \;+\frac{1}{\epsilon_{0}}\int d^{3}r\Pi^{\prime }\cdot P^{⊥}+\frac{1}{2\epsilon_{0}}\int d^{3}r(P^{⊥}{)}^{2}\\ & \;+\frac{1}{2M}\sum_{\alpha }\int \left\{P\cdot [\Theta_{\alpha }\times B]+[\Theta_{\alpha }\times B]\cdot P\right\}d^{3}r\\ & \;+\sum_{\alpha }(-1{)}^{\alpha +1}\frac{1}{2m_{\alpha }}\int \left(p\cdot [\Theta_{\alpha }\times B]+[\Theta_{\alpha }\times B]\cdot p\right)d^{3}r\\ & \;+\sum_{\alpha }\frac{{\left(\int d^{3}r\Theta_{\alpha }\times B\right)}^{2}}{2m_{\alpha }}. \end{array}$$

The integral in the third term of the square of the longitudinal polarization $P^{∥}$ contains the Coulomb energy of the system, and we may drop infinite self-energies [14]. The sum over α can be carried out in the first and the second terms. Also in the seventh term, the sum can be performed to yield dependence on the polarization P and with the help of the sum rule in equation (4.12), we have


(5.7) $$\begin{array}{lrlr} & H^{\prime } & =\frac{{𝐏}^{2}}{2M}+\frac{{𝐩}^{2}}{2\bar{m}}+\frac{e_{1}e_{2}}{4\pi \epsilon_{0}|𝐪|}+\frac{1}{2}\int d^{3}𝐫\left\{\frac{{\Pi^{\prime }}^{2}}{\epsilon_{0}}+\frac{{𝐁}^{2}}{\mu_{0}}\right\} & \\ & & \;+\frac{1}{\epsilon_{0}}\int d^{3}𝐫\Pi^{\prime }\cdot P+\frac{1}{2\epsilon_{0}}\int d^{3}𝐫(P^{⊥}{)}^{2} & \\ & & \;+\frac{1}{2M}\int d^{3}𝐫\left\{𝐏\cdot [P\times 𝐁]+[P\times 𝐁]\cdot 𝐏\right\} & \\ & & \;+\sum_{\alpha }(-1{)}^{\alpha +1}\frac{1}{2m_{\alpha }}\int d^{3}𝐫\left(𝐩\cdot [\Theta_{\alpha }\times 𝐁]+[\Theta_{\alpha }\times 𝐁]\cdot 𝐩\right) & \\ & & \;+\sum_{\alpha }\frac{{\left(\int d^{3}𝐫\Theta_{\alpha }\times 𝐁\right)}^{2}}{2m_{\alpha }}. & \end{array}$$

where we have set $\bar{m}$ to denote the reduced mass $\bar{m}=\frac{m_{1}m_{2}}{m_{1}+m_{2}}$. Equation (5.7) is the desired result giving the total Hamiltonian for a system of two charges exhibiting both internal and gross motions in interaction with electromagnetic fields. This result is exact within the non-relativistic framework of the original theory, but has considerable advantages for applications involving bound systems. The physical contents of the various terms in the Hamiltonian can now be discussed, considering them in turn.

### (a) Physical interpretation

The first term to be denoted $H_{c}$ is $\frac{{𝐏}^{2}}{2M}$. This is the zero-order Hamiltonian of the centre of mass M, position vector 𝐑, and canonical momentum 𝐏. It represents the Hamiltonian of a free particle. The eigenfunctions of $H_{c}$ are simply plane waves of momentum 𝐏=ℏ𝐊, and we can write


(5.8) $$\begin{array}{lcr} & H_{c}|𝐊\rangle =E(K)|𝐊\rangle ;\;\;\;E(K)=\frac{\hbar^{2}K^{2}}{2M}. & \end{array}$$

The coupling of the centre of mass to the fields then occurs only via higher multipoles as contained in the subsequent terms discussed as follows.

The second and third terms can be grouped together and denoted as $H_{p}$


(5.9) $$H_{p}=\frac{p^{2}}{2\bar{m}}+\frac{e_{1}e_{2}}{4\pi \epsilon_{0}|q|},$$

and the result can immediately be recognized as the Hamiltonian describing the internal motion of the hydrogen-like system including the effect of the finite mass of the system as contained in the effective mass $\bar{m}$. The eigenproblem is identical to the hydrogen-like system, and we can write


(5.10) $$\begin{array}{lcr} & H_{p}|i>=E_{i}|i\rangle , & \end{array}$$

where the label i denotes a set of quantum numbers that is sufficient to define the state as a spinless hydrogenic system.

The fourth term in the Hamiltonian pertains to the free electromagnetic field and is thus denoted as $H_{f}$


(5.11) $$\begin{array}{lcr} & H_{f}=+\frac{1}{2}\int d^{3}𝐫\left\{\frac{{\Pi^{\prime }}^{2}}{\epsilon_{0}}+\frac{{𝐁}^{2}}{\mu_{0}}\right\}. & \end{array}$$

Recall that $\Pi^{\prime }$ is the field canonical momentum conjugate to the vector potential. The eigenproblem for the free field Hamiltonian is well known and can thus be written at once. The single photon states of wavevector 𝐤 and polarization λ satisfy the equation


(5.12) $$\begin{array}{lcr} & H_{f}|𝐤,\lambda \rangle =\hbar \omega (k)|𝐤,\lambda \rangle . & \end{array}$$

The fifth term, denoted as $H_{int}^{\left(1\right)}$ , is recognized as the interaction of the entire electric multipole series with the field. We have


(5.13) $$\begin{array}{lcr} & H_{int}^{\left(1\right)}=\frac{1}{\epsilon_{0}}\int d^{3}𝐫\Pi^{\prime }\cdot P. & \end{array}$$

It will be seen shortly that the leading term of $H_{int}^{\left(1\right)}$ is the electric dipole term followed by the electric quadrupole terms and so on through all the higher multipole interaction terms. These interactions involve the coupling of the multipoles to the transverse displacement field evaluated at the centre of mass coordinate 𝐑 which thus enters only as a parameter, since all attributes of the charge system merely involve the internal motion.

The sixth term is a field-independent term that involves only the integral of the squared transverse electric polarization and is denoted as $H_{int}^{\left(2\right)}$


(5.14) $$\begin{array}{lcr} & H_{int}^{\left(2\right)}=\frac{1}{2\epsilon_{0}}\int d^{3}𝐫[P^{⊥}{]}^{2}. & \end{array}$$

This term is important in calculations of the Lamb shift [30,31] but does not influence any of the other manifestations of the interaction with electromagnetic field.

The seventh term, denoted as $H_{int}^{\left(3\right)}$, involves the gross motion. We have


(5.15) $$\begin{array}{lcr} & H_{int}^{\left(3\right)}=\frac{1}{2M}\int \left\{𝐏\cdot (P\times 𝐁)+(P\times 𝐁)\cdot 𝐏\right\}d^{3}𝐫. & \end{array}$$

As we explain later, this term is the origin of the Röntgen current [17], and on performing all space integration by parts, the term can be understood classically as a coupling between an electric field $\dot{𝐑}\times 𝐁$ generated by the centre of mass motion at velocity $\dot{𝐑}=𝐏/M$ in the magnetic field 𝐁. The interaction corresponds to the coupling of this electric field to the multipole polarization field P . The eighth term, denoted as $H_{int}^{\left(4\right)}$, affects primarily the internal motion


(5.16) $$\begin{array}{lcr} & H_{int}^{\left(4\right)}=\sum_{\alpha }(-1{)}^{\alpha +1}\frac{1}{2m_{\alpha }}\int \left(𝐩\cdot [\Theta_{\alpha }\times 𝐁]+[\Theta_{\alpha }\times 𝐁]\cdot 𝐩\right)d^{3}𝐫. & \end{array}$$

This corresponds to the coupling of the entire magnetic multipole series to the magnetic field. We can write


(5.17) $$\begin{array}{lcr} & H_{int}^{\left(4\right)}=-\int d^{3}𝐫M\cdot 𝐁, & \end{array}$$

where M is the magnetization field for the two-particle system


(5.18) $$\begin{array}{lcr} & M=-\sum_{\alpha }(-1{)}^{\alpha +1}\frac{1}{2m_{\alpha }}\left(\Theta_{\alpha }\times 𝐩-𝐩\times \Theta_{\alpha }\right). & \end{array}$$

The final term, denoted as $H_{int}^{\left(5\right)}$, is


(5.19) $$\begin{array}{lcr} & H_{int}^{\left(5\right)}=\sum_{\alpha }\frac{{\left(\int d^{3}𝐫\Theta_{\alpha }\times 𝐁\right)}^{2}}{2m_{\alpha }}. & \end{array}$$

This is referred to as the two-particle diamagnetic energy term which is reminiscent of the ${𝐀}^{2}/2m$ term in the conventional theory.

The above theory forms the basis for the application involving the radiative transitions of atoms and molecules, for example, the evaluation of emission and absorption processes, the London–van der Waals forces between two neutral atoms and the scattering of light from atoms and molecules. It became clear that in many such processes, the multipolar formulation is more advantageous than the conventional minimal coupling formulation [2,32].

### (b) Expansion in multipoles

The multipole series embodied in the definitions of P can be explicitly derived using the Taylor expansion of the delta function entering these expressions in powers of $\left({𝐪}_{\alpha }-𝐑\right)$. We have


(5.20) $$\begin{array}{lcr} & \delta \left\{𝐫-𝐑-\lambda ({𝐪}_{\alpha }-𝐑)\right\}=\left\{1-\lambda (q_{\alpha }-R{)}_{j}\frac{\partial }{\partial r_{j}}+........\right\})\delta (𝐫-𝐑). & \end{array}$$

Thus, we have for a component of P


(5.21) $$P_{i}=\sum_{\alpha }e_{\alpha }(q_{\alpha }-R{)}_{i}\int_{0}^{1}d\lambda \left\{1-\lambda (q_{\alpha }-R{)}_{j}\frac{\partial }{\partial r_{j}}+........\right\}\delta (r-R).$$

We may now perform the λ integration term by term, make use of equation (5.3) to write the result as follows:


(5.22) $$P_{i}=\left(d_{i}+Q_{ij}\frac{\partial }{\partial r_{j}}+......\right)\delta (r-R).$$

where $d_{i}$ is the ith component of the dipole moment vector , and $Q_{ij}$ is the quadupole tensor. Now consider the two-particle case and assume further that we are dealing with a neutral system $e_{1}=-e_{2}=e$ We have for the dipole moment vector


(5.23) $$\begin{array}{lrr} & 𝐝=\sum_{\alpha =1}^{2}e_{\alpha }({𝐪}_{\alpha }-𝐑)=e𝐪. & \end{array}$$

The quadrupole tensor can also be expressed in terms of components of the internal variable as follows:


(5.24) $$\begin{array}{lcr} & Q_{ij}=-\frac{1}{2}\sum_{\alpha }e_{\alpha }(q_{\alpha }-R{)}_{i}(q_{\alpha }-R{)}_{j}=-\frac{1}{2}eq_{i}q_{j}\left(\frac{m_{2}^{2}-m_{1}^{2}}{M^{2}}\right). & \end{array}$$

This exhibits finite mass effects contained in the factor between the brackets. In the limit when $m_{2}>>m_{1}$, this factor approaches unity, and we get the usual definition for the quadrupole tensor of hydrogen.

A similar expansion can be performed for the magnetic series which involves $\Theta_{\alpha }$. We write


(5.25) $$\begin{array}{lrlr} & \Theta_{\alpha } & =\sum_{\beta }e_{\beta }\int_{0}^{1}d\lambda \left(\lambda \delta_{\alpha \beta }-\frac{m_{\alpha }}{M}(\lambda -1)\right)({𝐪}_{\beta }-𝐑) & \\ & & \;\times \left\{1-\lambda ({𝐪}_{\beta }-𝐑).\nabla +\cdots \;.\right\}\delta \left(𝐫-𝐑)\right). & \end{array}$$

We are interested, however, in the leading order in the approximation in which we retain the dipole term and ignore all higher multipoles. We therefore obtain for the electric polarization in the dipole approximation


(5.26) $$\begin{array}{lcr} & P\approx 𝐝\delta (𝐫-𝐑), & \end{array}$$

and the corresponding approximation for $\Theta_{\alpha }$ turns out to be


(5.27) $$\begin{array}{lrlr} & \Theta_{\alpha } & \approx \frac{1}{2}e𝐪\delta (𝐫-𝐑) & \\ & & =\frac{1}{2}𝐝\delta (𝐫-𝐑);\;\;\;\;\alpha =1,2. & \end{array}$$

The magnetic polarization then becomes


(5.28) $$\begin{array}{ll} M & =\frac{1}{2}\sum_{\alpha }\frac{(-1{)}^{\alpha +1}}{m_{\alpha }}\left\{\Theta_{\alpha }\times p-p\times \Theta_{\alpha }\right\}\\ & =\frac{e}{4}\left(\frac{1}{m_{1}}-\frac{1}{m_{2}}\right)(q\times p-p\times q)\delta (r-R)\\ & =\mu \delta (r-R), \end{array}$$

where μ is the magnetic dipole moment


(5.29) $$\mu =\frac{1}{4}e\left(\frac{1}{m_{1}}-\frac{1}{m_{2}}\right)\left(q\times p-p\times q\right).$$

Another term that can be reduced to its dipole approximation form is the field-independent interaction $H_{int}^{\left(2\right)}$


(5.30) $$\begin{array}{lcr} & H_{int}^{\left(2\right)}=\frac{1}{2\epsilon_{0}}\int d^{3}𝐫[P^{⊥}{]}^{2}. & \end{array}$$

This interaction term was analysed by Power & Zienau [1] and shown to give rise to a singular contribution to the non-relativistic Bethe term of the Lamb shift when the dipole coupling term $𝐝.\Pi^{\prime }/\epsilon_{0}$ is used.

The dipole approximation affects various other terms in the multipolar Hamiltonian and has implications for both the translational and the internal motions. The presence of the delta function δ(𝐫−𝐑) in P and $\Theta_{\alpha }$ in the dipole approximation leads to immediate evaluations of the space integrals. The Hamiltonian reduces the dipole approximation to


(5.31) $$\begin{array}{lrlr} & H^{\prime } & =\frac{{𝐏}^{2}}{2M}+\left(\frac{{𝐩}^{2}}{2\bar{m}}-\frac{e^{2}}{4\pi \epsilon_{0}|𝐪|}\right)+\frac{1}{2}\int d^{3}𝐫\left\{\frac{{\Pi^{\prime }}^{2}}{\epsilon_{0}}+\frac{{𝐁}^{2}}{\mu_{0}}\right\} & \\ & & \;+\frac{1}{\epsilon_{0}}𝐝\cdot \Pi^{\prime }(𝐑) & \\ & & \;+\frac{1}{2M}\left\{𝐏\cdot [𝐝\times 𝐁(𝐑)]+[𝐝\times 𝐁(𝐑)]\cdot 𝐏\right\} & \\ & & \;-\mu \cdot 𝐁(𝐑)+\frac{[𝐝\times 𝐁(𝐑){]}^{2}}{8\bar{m}} & \\ & & \;+\frac{1}{2\epsilon_{0}}\int d^{3}𝐫(P^{⊥}{)}^{2}. & \end{array}$$

The result given in equation (5.31) is the non-relativistic Hamiltonian for a system of two bound charged particles in interaction with the radiation field. In this Hamiltonian, the internal motion of the charge system is clearly distinguished from the gross motion. It is seen that the zero-order Hamiltonians of the three subsystems (gross motion, internal motion and fields) are exactly separated off and given by the first three terms occupying the first line of equation (5.31). The rest of the terms represent the interaction in which the three subsystems are coupled. The $𝐝\cdot \Pi^{\prime }(𝐑)/\epsilon_{0}$ term represents the coupling of the dipole moment to the field $\Pi^{\prime }$, which is interpreted as the electric displacement field, and in field quantization, it satisfies equation (3.19) as the usual commutation relation [27]. The next term is the Röntgen interaction which we discuss further below. The next term is the magnetic dipole interaction, and the penultimate term is a diamagnetic-type energy which is relatively small. The last term has been rewritten in its original form involving the integral of the square of the polarization. It is a self-interaction term which is known to contribute to the Lamb shift [2].

There is an effective Hamiltonian $H_{d}$ used in the literature, defined as the version of $H^{\prime }$ in the electric dipole approximation, and is restricted to the following truncated form [33–35]:


(5.32) $$\begin{array}{lcr} & H_{d}=\frac{{𝐏}^{2}}{2M}+H_{a}+H_{f}+\frac{1}{\epsilon_{0}}𝐝\cdot \Pi^{\prime }(𝐑)+\frac{1}{2M}\left(𝐏.𝐝\times 𝐁(𝐑)+𝐝\times 𝐁(𝐑).𝐏\right), & \end{array}$$

where $H_{a}$ is the hydrogen-like Hamiltonian describing the internal motion while $H_{f}$ is the field Hamiltonian, and as far as interactions are concerned, we may replace $\Pi^{\prime }$ with $-\epsilon_{0}𝐄$.

Equation (5.32) is the starting point for the investigation of gross motion effects. We note in particular the appearance of the **P**-dependent terms, which we refer to as the Röntgen interaction. Note that this is the dipole approximation of the full form in equation (5.15) where the entire set of multipoles enter. For instance, one can talk about a quadrupole Röntgen interaction, etc.

The leading Röntgen effect involves an electric dipole moment 𝐝, which is assumed moving in the presence of a magnetic field. We refer to the Röntgen interaction in equation (5.32) as $H_{R}$ and write it in a simpler form by making use of Maxwell’s equation ∇⋅𝐁=0,


(5.33) $$\begin{array}{lcr} & H_{R}=\frac{𝐝\times 𝐁\cdot 𝐏}{M}. & \end{array}$$

A second effect due to translational motion is the Aharonov–Casher (AC) effect [22,36]. Here a particle carrying a magnetic dipole moment μ is assumed to be moving in the presence of an electric field 𝐄. The AC effect is, in fact, the electric form of the Röntgen effect, and its form can be obtained by replacing the 𝐝 by μ/c and 𝐁 by 𝐄/c. We have


(5.34) $$H_{AC}=\frac{\mu \times E\cdot P}{Mc^{2}}.$$

Both the Röntgen effect and the AC effect manifest themselves in phase shifts of the Aharonov–Bohm kind. Although derived here from the Röntgen interaction by appeal to electromagnetic symmetry, it was Horsley *et al.* [26] who managed to provide a rigorous derivation based on the Lagrangian formalism and by introducing the concept of centre of mass-energy 26,35, which led to the appearance of the AC interaction term along with other familiar interaction terms in the multipolar formulation and which conforms with equation (5.34) in the magnetic dipole approximation.

The phase phenomena associated with moving electric and magnetic dipoles have been referred to as the He–McKellar–Wilkens (HMW) topological phase which was predicted by He and McKellar [37] and by Wilkens [20] and has been experimentally investigated [25]. The first topological phase stems from the Röntgen interaction. This can be written in the Aharanov–Bohm interaction form [38] as follows:


(5.35) $$\begin{array}{lcr} & H_{R}=e{𝐯.𝐀}_{R}, & \end{array}$$

where 𝐯=𝐏/M and ${𝐀}_{R}$ is an effective Röntgen vector potential


(5.36) $$\begin{array}{lcr} & {𝐀}_{R}=\frac{1}{e}𝐝\times 𝐁(𝐑). & \end{array}$$

This indicates an Aharonov–Bohm-type effect while the particle is travelling along a closed path C, associated with the vector potential ${𝐀}_{R}$. We have


(5.37) $$\begin{array}{lcr} & \Delta S_{R}/\hbar =\frac{1}{e\hbar }\oint_{C}(𝐝\times 𝐁)\cdot 𝐝𝐥. & \end{array}$$

The Aharonov–Casher effect can be treated along the lines above. The effective Aharonov–Casher vector potential is


(5.38) $$A_{AC}=\frac{1}{c^{2}}\mu \times E.$$

Instead of the Röntgen phase shift (5.39), we obtain the Aharonov–Casher phase shift


(5.39) $$\Delta S_{AC}/\hbar =\frac{1}{c^{2}\hbar }\oint_{C}(\mu \times E)\cdot dl.$$

The AC effect has already been observed experimentally [39,40].

## 6. Comments and conclusions

In this article, we sought to highlight the roles of gauge and canonical transformations when bound neutral atomic or molecular systems interact with electromagnetic fields. There is in fact a third equivalent transformation, namely the addition of a total time derivative to the Lagrangian. The equivalence of this method to the gauge transformation method has been succinctly verified in the conclusions section of [14]. The main feature of the PZW theory is that the interaction between electromagnetic fields and a bound system of charges arises in terms of multipoles, both electric and magnetic, and as a result, the coupling of the multipolar sources is to the field intensities 𝐄 and 𝐁. This should be contrasted with the case of the conventional theory where the coupling is between the charge and current densities with gauge-dependent electromagnetic potentials 𝐀 and Φ. The electric polarization and the magnetization enter as closed integral forms leading to quantum optics in the most usable form in terms of electric and magnetic dipoles and quadrupoles and, in principle, to any multipolar order, interacting with gauge-invariant electric and magnetic fields. The theoretical framework involving the canonical transformation in effect follows the formalism first derived by Power & Zienau [1] and generalized to the Power–Zienau–Woolley (PZW) formalism with contributions by Woolley on the same subject [10–13]. A significant extension included the gross motion of matter in addition to the internal motion and a further extension included the many centres which allows for inter- as well as intra-atomic and molecular processes [41]. The procedure that has led to the same PZW Hamiltonian and which involved a gauge transformation is less familiar. A recent review by Stokes & Nazir [42] discusses gauge invariance in non-relativistic QED and points out how gauge ambiguities can arise in that context.

The formalism here has drawn attention to some features of significant current interest, most notably the Röntgen interaction and the Aharonov–Casher interaction, both of which were initially ignored as small effects. However, including the Röntgen interaction has been shown to account for the difference between canonical and mechanical momenta in the electrodynamics of dielectrics, and the effect is predicted to lead to additional forces acting on a moving atom [34,43]. It must also be taken into account in order to exhibit the time dilation effect in the evaluation of the spontaneous emission of such a moving atom [44–46]. It is interesting to note that the AC effect has already been observed experimentally [39,40], but as far as the author knows, there is no direct experimental confirmation yet for the Röntgen effect which followed Wilkins’ suggestion [21]. Both effects are predicted to lead to quantum phase effects of the Aharonov–Bohm kind. The phase associated with the Röntgen interaction is now referred to by some as the He–Mckellar–Wilkins phase. Recent work has extended the validity of the AC interaction, equation (5.34) to any particle carrying a magnetic dipole, where the magnetic moment is attributable to the spin angular momentum such as in the case with neutrons and electrons [36,40,47]. It seems reasonable to suggest that, similarly, some real particle endowed with a permanent electric dipole moment moving in a magnetic field should exhibit the Röntgen phase. Unfortunately, an atom does not possess a permanent dipole moment, so only an induced dipole moment can be used, as done in the experiment [25]. Furthermore, there is at present no known elementary particle endowed with a permanent electric dipole moment. However, in 2011, a substantial permanent dipole moment was reported, carried by a homonuclear rubidium molecule [48], and there is also another report of a permanent dipole moment associated with NiO [49].

## Data Availability

This article has no additional data.

## Dedication

The author wishes to dedicate this article in memory of Professor Rodney Loudon who was one of the most influential theoretical physicists, internationally, in the fields of quantum optics and condensed matter.
